# Adrenal Haemorrhage in the Context of Acute Systemic Illness: Three Cases With Diverse Clinical Presentations

**DOI:** 10.7759/cureus.99554

**Published:** 2025-12-18

**Authors:** Nowrin Jahan Arvy, Tanweer Ahmed

**Affiliations:** 1 Acute Medicine, Milton Keynes University Hospital, Milton Keynes, GBR; 2 Cardiology, Milton Keynes University Hospital, Milton Keynes, GBR

**Keywords:** adenoma, adrenal adenoma, adrenal haemorrhage, adrenal insufficiency (ai), essential thrombocythaemia, leucocytosis, pulmonary embolism, waterhouse-friderichsen

## Abstract

Adrenal haemorrhage (AH) is uncommon and often underrecognized and may complicate severe systemic stress, infection, or coagulopathy. Prompt diagnosis is essential to exclude adrenal insufficiency and adrenal neoplasms. Three cases of AH occurring in varied clinical settings describe diagnostic approaches, management, and outcomes and draw insights for clinicians. Case 1 is a patient in septic shock hospitalised in the ICU who developed bilateral AH (Waterhouse-Friderichsen syndrome). Case 2 is about a patient admitted for chest pain, fall, and suspected infective exacerbation of chronic obstructive pulmonary disease (COPD), later found to have unilateral AH in the context of pulmonary embolism. Case 3 indicates a patient with essential thrombocythaemia and hypertension, presenting with acute abdomen after a flu-like illness, found to have a left adrenal lesion with features of haemorrhage within a lipid-rich adenoma. In all three cases, adrenal insufficiency was assessed; in two cases, steroid therapy was administered, while in one (Case 2), it was not required. Repeat imaging over months showed resolution or stability without malignant transformation. These three cases underscore the heterogeneity of AH presentations, the need for prompt endocrine evaluation, imaging follow-up, and multidisciplinary care. Recognition in atypical settings (e.g., sepsis, respiratory infection, and haematological disease) is key to preventing adrenal crisis and guiding management.

## Introduction

Adrenal haemorrhage (AH) is a rare but clinically significant phenomenon that can lead to life-threatening adrenal insufficiency if bilateral or extensive [1]. It is often secondary to severe physiological stress, sepsis, trauma, coagulopathy, or anticoagulation therapy but may also occur in patients without overt risk factors [2]. Our objective is to illustrate how systemic triggers and underlying conditions influence clinical presentation, endocrine evaluation, and follow-up. In clinical practice, the symptoms presented by AH exhibit diverse patterns, and they can be associated with adrenal insufficiency or organ failure in multiple organs. Nevertheless, in many patients, the clinical course of AH can remain asymptomatic. Unilateral AH is frequently an incidental radiologic finding on imaging performed for other reasons [2,3].

The pathophysiology involves a unique vascular dam architecture, which increases the predisposition of the adrenal gland to bleed. It includes adrenal vein thrombosis or venous congestion under situations of high cortisol demand or vascular stress, culminating in haemorrhagic infarction leading to rupture in tiny and frail adrenal venules [4]. Diagnostically, distinguishing pure haemorrhage from haemorrhage into an underlying adrenal neoplasm is critical, since management and prognosis differ. Magnetic resonance imaging (MRI) and follow-up imaging are valuable in differentiation [5]. Furthermore, assessment for adrenal insufficiency (via cortisol levels and dynamic testing) is essential to guide therapy.

Here, we present three patients with AH encountered in different clinical settings: septic shock, respiratory disease with pulmonary embolism, and pre-existing haematological disease with varying management strategies and outcomes. We aim to highlight lessons for frontline clinicians. 

## Case presentation

Case 1

Presentation and Clinical Course

A 64-year-old female (case demographics) was admitted to the intensive care unit (ICU) with fulminant septic shock secondary to pneumonia. This was her third presentation to the emergency department within a span of several days with an unresolved chest infection with repeated courses of antibiotics. On presentation, she was profoundly hypotensive despite fluid resuscitation, hypothermic, and exhibited altered mental status (Table 1).

**Table 1:** Vitals on admission Observations: temperature, blood pressure, respiratory rate, and oxygen saturation in the first few hours of admission

Time	Temperature (°C)	Temperature location	Heart rate (bpm)	Pulse regularity	Pulse location	Respiratory rate (br/min)	Respiratory distress	SBP/DBP (mmHg)	Mean arterial pressure (mmHg)	SpO2 (%)	SpO2 location	Oxygen therapy	Oxygen flow rate (L/min)	Capillary refill
16:30 GMT	36	Tympanic	81	Regular	Right arm	18	None	75/54	61	96	Right hand	Nasal cannula	1	Greater than 3 sec
15:00 GMT	35.8	Tympanic	80	Regular	Right arm	18		75/48	57	94	Left hand	Nasal cannula	1	Greater than 3 sec
14:00 GMT	35.8	Tympanic	82	Regular	Right arm	14	None	75/45	55	95	Left hand	Nasal cannula	1	Greater than 3 sec
12:49 GMT	35.7	Tympanic	80	Regular	Right arm	16		80/47	58	94	Left hand	Nasal cannula	1	Greater than 3 sec

Laboratory markers showed elevated C-reactive protein (CRP), procalcitonin, and deranged electrolytes and clotting parameters (Table 2).

**Table 2:** Blood test results on admission The most relevant blood tests on admission. High procalcitonin indicates sepsis, possibly due to bacterial infection.

​Parameter	Value	Reference
Hb​	101​	Normal range (typically 120–160 g/L)
WCC​	4.3​	Normal ~4–11 ×10⁹/L
Clotting profile ​	Normal​	
Na​	127 ​(low)	Normal ~135–145 mmol/L
Potassium	4.6	Normal 3.5-5.3 mmol/L
Bi-carb	16.1 (low)	Normal 22-29 mEq/L
Creatinine ​	209 (AKI 3)​	Normal 49-90 umol/L
ALP ​	307​	Normal 30-130 IU/L
CRP ​	221​	Normal 0-6 mg/L
Procalcitonin	7.53​ (high)	Normal 0-0.4 nanogram/ml

**Figure 1:**
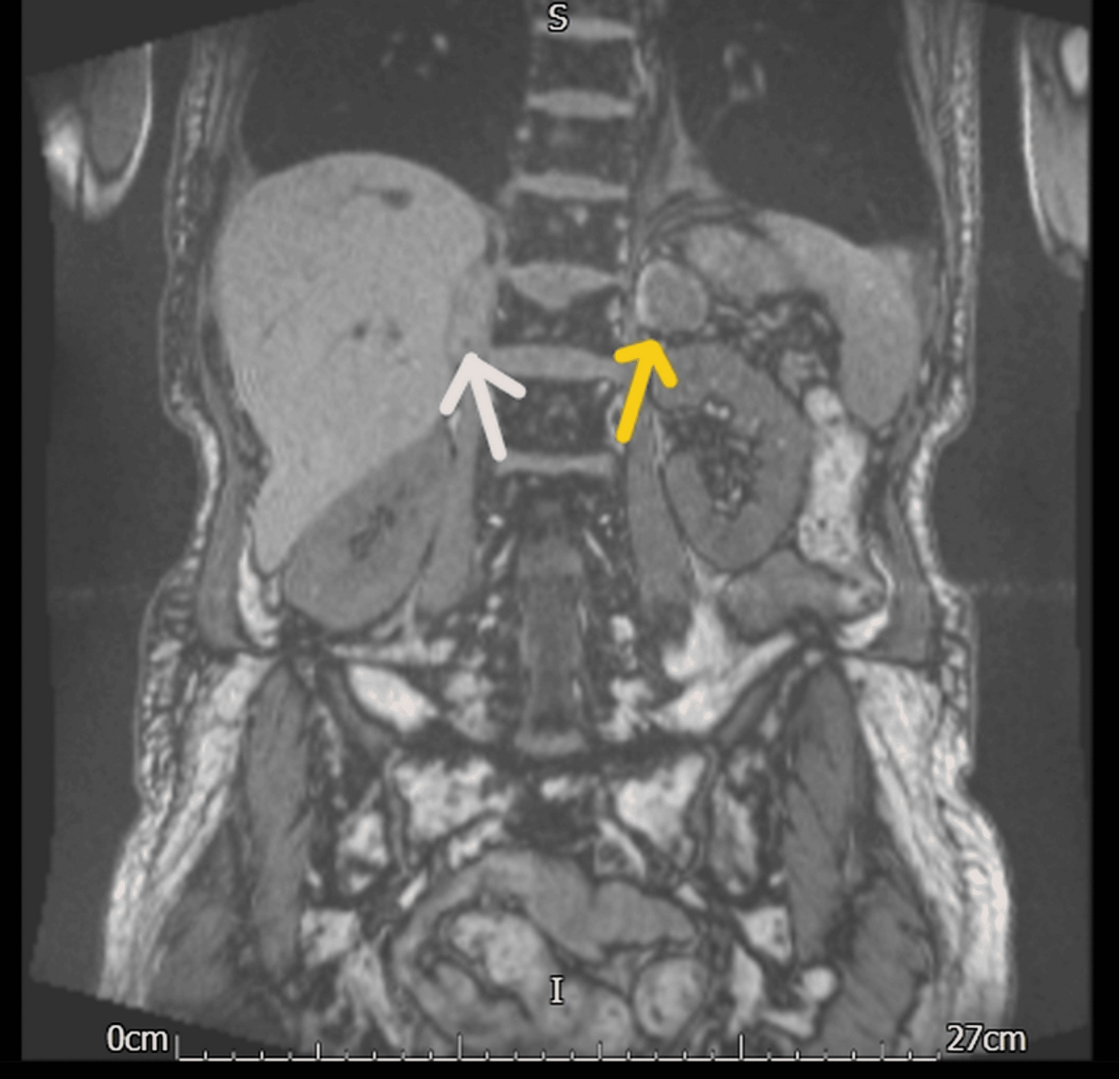
MRI of the adrenal glands MRI adrenal gland (coronal T1 weighted) is showing both adrenal glands are enlarged, the left one (the yellow arrow indicating the left adrenal gland) being larger. Both glands are ellipsoid/rounded in shape rather than the typical bifid appearance. The left adrenal gland measures a maximum size of 38 mm; the right adrenal gland (the pink arrow is showing the right adrenal gland) measures approximately 27 mm in maximum diameter.

Despite broad-spectrum antibiotics and fluid resuscitation, her condition was progressively deteriorating; therefore, steroids were initiated in view of high suspicion of adrenal insufficiency due to refractory hypotension and hyponatremia. She was closely monitored in the ICU but did not need a vasopressor. Contrast-enhanced CT of the abdomen followed by MRI (Figure 1) revealed bilateral adrenal enlargement with heterogeneous high attenuation consistent with acute bilateral AH.

Endocrine Evaluation

Morning cortisol was low (44 nmol/L, normal value: 275-555 nmol/L). The electrolyte panel demonstrated hyponatraemia and episodes of hypoglycaemia. A diagnosis of primary adrenal crisis was made in the setting of bilateral AH (i.e., Waterhouse-Friderichsen syndrome).

Management

The patient was started promptly on intravenous hydrocortisone (initially 100 mg and then 50 mg QDS) along with intravenous antibiotics. She was in the ICU for two to three days with close monitoring and intravenous fluid and supportive care.

Outcome and Follow-up

Over several days, the patient gradually stabilised, and she was weaned off support. She was transitioned to oral hydrocortisone and fludrocortisone, with endocrine follow-up. On long-term follow-up, adrenal function remained impaired (after five months, 9 AM cortisol was less than 100 nmol/L, aldosterone <60 pmol/L with renin 1.6 ng/ml/hr while on oral hydrocortisone), necessitating ongoing replacement therapy. 

Summary (Case 1):

A classical presentation of bilateral AH in septic shock (Waterhouse-Friderichsen syndrome) [3], causing adrenal crisis. Early recognition and steroid replacement were critical to survival [4].

Case 2

Presentation and Clinical Course

A 72-year-old man with severe chronic obstructive pulmonary disease (COPD), hypertension, osteoporosis, and prior subarachnoid haemorrhages presented after an unwitnessed fall. He reported left-sided chest pain from the evening before and was later found on the floor, having been unable to summon help. He had no memory of the event. He had a background of chronic hyponatremia and was being treated for an infective exacerbation of COPD with antibiotics and regular nebuliser. Initial blood test showed raised white cell count (WCC) and CRP (Table 3).

**Table 3:** Blood test results on admission

Parameter	Value	Reference / note
Hemoglobin	152	Normal range (typically 120–160 g/L)
White cell count	21.1	Very high (normal ~4–11 ×10⁹/L)
Sodium	129	Low (normal ~135–145 mmol/L)
Potassium	3.8	Normal (3.5–5.0 mmol/L)
C-reactive protein	182	High (normal <10 mg/L)

Clinical Findings

Initial workup excluded acute coronary syndrome. The patient was started on levofloxacin for a respiratory infection. CT chest showed secretions in the left main bronchus, prompting bronchoscopy, which showed no endobronchial lesions.

On day 3, a CT pulmonary angiogram (CTPA) to evaluate dyspnoea and chest pain revealed bilateral pulmonary embolism and a new 34 mm well-defined low-attenuation mass at the site of the previously demonstrated left adrenal gland, most likely representing haemorrhage into the adrenal gland.

**Figure 2:**
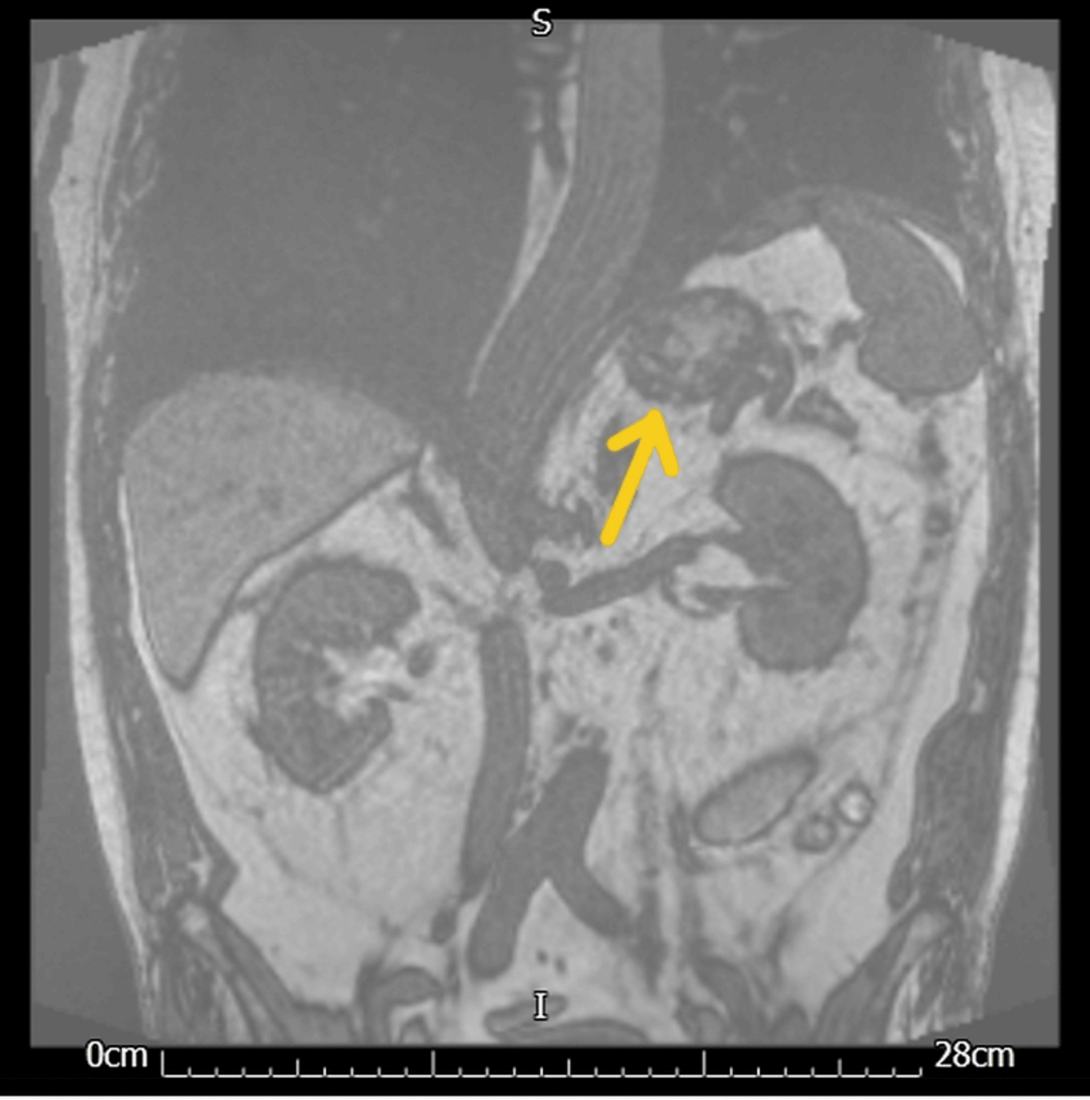
MRI adrenal glands following the CT pulmonary angiogram (CTPA) MRI adrenal gland (coronal T1-weighted phase 1): the yellow arrow shows the lesion involving the left adrenal gland identified, which measured 32 x 31 mm and showed no significant interval change in comparison to a previous study.

An MRI (Figure 2) was done in three days' time, which identified a lesion involving the left adrenal gland; it measured 32 x 31 mm and showed no significant interval change in comparison to the previous study. In the given clinical setting, the most likely possibility was haemorrhage in the left adrenal gland.

Endocrine Evaluation

He had mild hyponatremia but normal potassium and glucose levels. Morning cortisol was 579 nmol/L, which is within the normal range, thereby excluding overt adrenal insufficiency. Given stable hemodynamics and the absence of adrenal crisis, the patient was managed conservatively.

Management

The patient was initiated on intravenous antibiotics (IV Tazocin) and fluids, received supplemental oxygen, and was started on apixaban for the pulmonary embolism. He did not need any steroids. 

An F/U MRI performed approximately three months later confirmed that the left adrenal gland appears much smaller and measured 21 x 13 mm in comparison to 32 x 31 mm in the previous study. A normal right adrenal gland was seen.

Outcome and Follow-up

The patient’s respiratory status gradually improved, although baseline respiratory function declined over time, and he was commenced on home non-invasive ventilation (NIV). An endocrinology follow-up was arranged, with a scheduled repeat MRI in 6 months for adrenal surveillance. 

Summary (Case 2)

A case of unilateral AH in a patient with chest infection and pulmonary embolism. There was no requirement for medical or surgical management for the AH as there was no clinical or biochemical sign of adrenal insufficiency.

Case 3

Presentation and Clinical Course

A 42-year-old man came with acute diffuse abdominal pain after two days of flu-like symptoms (fever, malaise, and myalgia) on the background of essential thrombocythemia (JAK2-positive) and had been taking aspirin for that. He was diagnosed with essential thrombocythemia about three years ago, incidentally, when he was admitted to the hospital with COVID pneumonitis and was started on aspirin from the haematology team. In this admission, his onset of abdominal pain was abrupt and moderate in severity (scoring 6/10), associated with nausea, lethargy, decreased oral intake, and altered bowel movements without any urinary symptoms. 

His clinical findings are febrile (39.7 °C) with normal blood pressure and normal saturation on room air (Table 4).

**Table 4:** Vitals on admission BP: blood pressure, MAP: mean arterial pressure, ACVPU: Alert, Confusion, Voice, Pain, and Unresponsive

Time (GMT)	Temp (°C)	Temp location	Heart rate (bpm)	Respiratory rate (breaths/min)	BP (mmHg)	BP position	MAP (mmHg)	SpO₂ (%)	O₂ therapy	ACVPU
00:24	39	Tympanic	127	24	150/82	Sitting	95	95%	Room air	A-Alert
00:30	39.7	Tympanic	111	22	142/78	Sitting	96	96%	Room air	A-Alert
05:42	37.9	Tympanic	101	16	125/74	Sitting	100	97%	Room air	A-Alert
09:18	37.5	Tympanic	94	15	120/71	Sitting	87	97%	Room air	A-Alert
10:24	38.2	Tympanic	91	18	145/88	Sitting	98	98%	Room air	A-Alert
13:00	38.8	Tympanic	91	18	145/88	Sitting	98	98%	Room air	A-Alert
15:03	38.2	Tympanic	91	18	145/88	Sitting	98	97%	Room air	A-Alert
17:00	38.8	Tympanic	91	18	145/88	Sitting	98	97%	Room air	A-Alert
18:11	38.2	Tympanic	91	18	145/88	Sitting	98	98%	Room air	A-Alert

Initial blood test showed raised CRP and neutrophils. He was found to be positive for influenza A on nasal swab PCR (Table 5).

**Table 5:** Blood test results on admission

Test	Result	Reference / note
Hemoglobin (Hb)	133 g/L	Normal (130-170 g/L)
White cell count (WCC)	10.2 x 10⁹/L	Normal (4.0–11.0 × 10⁹/L)
Platelets	511 x 10⁹/L	High (150–400 × 10⁹/L)
Lymphocytes	0.3 x 10⁹/L	Low (1.0–4.0 × 10⁹/L)
Neutrophils	9.2 x 10⁹/L	High (2.0–7.5 × 10⁹/L)
Sodium	136 mmol/L	Normal (135–145 mmol/L)
Potassium	2.6	Low (3.5–5.0 mmol/L)
C-reactive protein (CRP)	42	Elevated (<6)
GeneXpert influenza A RNA PCR	Detected	Positive for influenza A
Blood culture	Negative	No growth

A non-contrast CT was performed to r/o acute abdomen as a cause of persistent abdominal pain, which revealed a soft tissue lesion in the left upper quadrant between the pancreas, spleen, and kidney. To further characterise, a contrast CT of the chest, abdomen, and pelvis (CT TAP) was performed, which demonstrated an enhancing nodule/mass in the left adrenal region with adjacent ill-defined soft tissue, raising concern for adrenal pathology, including haemorrhage or infection as the likely cause of abdominal pain.

Endocrine and Infectious Work-up

Endocrine testing included cortisol, ACTH, renin, and aldosterone levels; infectious screens included TB, malaria, and other opportunistic pathogens. Plasma metanephrines were normal. Aldosterone was 380 pmol/L (normal range 90-700), renin activity 5.2 nmol/L/hr (elevated relative to reference 0.5-3.5), giving an aldosterone/renin ratio of 73 (<80: not consistent with primary aldosteronism) (aldosterone blood test was taken in the lying position, and the patient was on his regular losartan for hypertension at that time).

Management

The patient was started on intravenous antibiotics and Tamiflu (oseltamivir for influenza A). Potassium replacement for hypokalaemia and supportive care. He did not need any medical/surgical treatment for that AH as there was no hormonal insufficiency.

MRI scanning following discharge demonstrated a 2.7 × 2.5 cm lipid-rich left adrenal nodule showing internal haemorrhagic change but stable in appearance, consistent with haemorrhage into an adenoma rather than an aggressive neoplasm.

Outcome and Follow-up

During the endocrine clinic review, the patient was asymptomatic. A repeat MRI at six months later showed stability, no interval growth, and no evidence of malignancy. An MRA of renal arteries was scheduled due to elevated renin levels as per guidelines, which showed no stenosis, excluding secondary hyperaldosteronism. He was followed up in the outpatient endocrine clinic for hormonal surveillance. 

Summary (Case 3)

This case describes a unilateral AH in a patient presenting with flu-like symptoms (influenza A-positive) on a background of essential thrombocythemia. The haemorrhage occurred within the adrenal adenoma. Conservative management, endocrine assessment, and serial imaging confirmed a benign, non-functioning lesion that remained stable over time

## Discussion

The spectrum of presentations of AH is underscored by the varied precipitants, laterality, and underlying diseases in these three illustrative cases. Each case demonstrates the subtle balance between vascular stress, hemodynamic injury, and vulnerability of adrenal microcirculation. 

First, the precipitating stressor is frequently the onset event. In Case 1, overwhelming sepsis and hypotension most likely triggered adrenal infarction and hemorrhagic necrosis bilaterally, leading to a fulminant adrenal crisis in the style of the typical Waterhouse-Friderichsen syndrome [6], which is evidently found in the literature. Sepsis and endotoxemia are established precipitants of bilateral AH by way of endothelial damage, coagulopathy, and thrombosis of the adrenal veins in susceptible microvessels [7].

In Case 2, a more focused vascular insult potentially stemming from hypoxia, elevated venous pressure, or microthrombi associated with pneumonia, immobility, and pulmonary embolism might have exceeded the perfusion capacity of one adrenal gland, resulting in a unilateral haemorrhage. In Case 3, the intricate interaction of existing vascular susceptibility (possibly attributable to an underlying hematologic condition), systemic infection, and antiplatelet treatment may have made a pre-existing adenoma vulnerable to intratumoral bleeding. This occurrence of haemorrhage within an adrenal lesion is uncommon yet acknowledged, and the academic literature outlines instances of AH in relation to adenomas, carcinomas, and pheochromocytomas.

Indeed, unilateral haemorrhage predominates in clinical settings; the lateralisation pattern has a profound impact on the clinical result. With bilateral adrenal disease, the secretion of cortisol and aldosterone is lost or severely impaired, such that biochemical adrenal insufficiency results, and often shock if not treated immediately. By contrast, the unilateral haemorrhage frequently leaves the patient with enough preserved residual adrenal function in many cases so that the patient remains asymptomatic or has gentle symptoms [2,8], such as in Cases 2 and 3. 

The likelihood of a preexisting adrenal lesion (especially in Case 3) creates a diagnostic problem. Haemorrhage that has taken place within adenomas or other adrenal tumours has been reported. In the assessment of a hemorrhagic adrenal mass, it is imperative to differentiate between a pure hematoma and haemorrhage within a neoplastic structure [9]. Characteristics indicative of a benign adenoma encompass spontaneous regression over time, the absence of interval growth, a homogeneous internal structure observed in follow-up imaging, a lack of concerning enhancement noted on MRI, and the absence of biochemical hypersecretion. In contrast, imaging features such as irregular enhancement, interval growth, heterogeneous signalling extending beyond the phases of hemorrhagic decay, or evidence of functional hormone hypersecretion should heighten the suspicion of malignancy or pheochromocytoma. The application of MRI, particularly with fat-saturated and in-/out-of-phase sequences, can facilitate the differentiation of hemorrhagic signal characteristics and identify residual lesions beyond the haemorrhage itself. 

Diagnostically, imaging and endocrine assessment must occur in parallel. Computed tomography frequently serves as the first imaging technique in acute presentations and will sometimes indicate the presence of a hyperdense adrenal lesion in the initial stage; the attenuation lowers over time, such that the appearance shifts. MRI also provides temporal signal mapping of haemorrhage (acute, subacute, and chronic phases) and facilitates differentiation of haemorrhage from enhancing tumour [10]. 

Endocrine testing also should include morning cortisol, ACTH, renin-aldosterone levels, and (as appropriate) metanephrines or catecholamines [11]. Morning cortisol level is a biochemical screening test for primary adrenal insufficiency. Low or intermediate morning cortisol level requires biochemical confirmation with a cosyntropin stimulation test. In contrast with morning cortisol level, hypercortisolism is diagnosed when a patient tests positive on at least two out of three screening biochemical tests (11 PM salivary cortisol, low-dose dexamethasone suppression test or 24-hour urinary free cortisol measurement).

For a suspected AH and adrenal crisis, glucocorticoid therapy need not be postponed until dynamic testing. For a unilateral or lower-risk tumour, safe endocrine testing and close follow-up are permissible. Interval imaging by three to six months (earlier if clinical concerns are suspected) is necessary to document regression or stability of the nodule; inability to regress or growth indicates further testing or surgical referral [12].

Therapeutically, the cornerstone is supportive care of the causative insult (e.g., thrombosis, infection, hemodynamic stabilisation), electrolyte and volume normalisation, and appropriate thrombosis prophylaxis when not contraindicated. For patients with adrenal insufficiency or in crisis (as in Case 1), high-dose glucocorticoid replacement (nearly always hydrocortisone) is life-saving and needs to be implemented urgently.

Mineralocorticoid replacement will become an option thereafter in the recovery stage. With patients having no biochemical adrenal failure and stable haemodynamics, conservative (non-interventional) care is reasonable with close monitoring. Surgical intervention is seldom required for nontraumatic AH but is limited for haemorrhage with mass effect, risk for expansion or rupture, or suspected underlying malignancy. In rare tumour-bleed cases (e.g. myelolipomas), transarterial embolisation has safely controlled bleeding without the assistance of open surgery [13]. 

Several limitations also limit the efficiency of generalising the results from our cases, mainly due to the retrospective design and small sample size. Moreover, none of the lesions received adrenal biopsy or surgical histopathology verification; the distinction between a pre-existing adenoma versus hemorrhagic pseudo-mass relies on imaging studies and clinical course rather than tissue verification. Lastly, the timing of imaging follow-up studies differed between cases and may limit the comparison of the temporal patterns of regression in a direct manner. From our experience and the wider literature, a number of important lessons are revealed. In the critically ill patient with unexplained refractory hypotension, electrolyte disorders, or abdominal imaging evidence of an adrenal mass(es), clinicians need to have a high index of clinical suspicion for AH. Cortisol measurement and empiric steroid treatment, when the index of suspicion is high, need not await definitive testing. In the non-crisis presentations, a dual imaging (CT and MRI) plus endocrinologic evaluation approach is required in order to distinguish haemorrhage from neoplasm [14]. Serial imaging follow-up (usually every three to six months) is required to establish regression or stability, and any lesion that does not regress needs to be closely evaluated for possible neoplastic conversion.

Lastly, intensivist-endocrinologist-radiologist-haematologist-surgeon collaboration is crucial in optimising decisions and excluding unnecessary intervention. In a nutshell, AH in clinical practice encompasses the whole range, from a life-threatening bilateral adrenal crisis to incidental bleeds in predisposing lesions. Awareness of the precipitants, judicious diagnostic work-up, and prudent collaborative management led by the clinical scenario may enable tailoring of the therapy, avoid overtreatment, and achieve optimum results.

## Conclusions

These case studies refer to three distinct presentations of AH in the context of systemic stress: the first as a complication of septic shock (also referred to as Waterhouse-Friderichsen Syndrome), the second as a complication of pulmonary embolism and severe chest infection, and the third as haemorrhage in a pre-existing adrenal adenoma in a patient with an underlying platelet disorder(thrombocythemia). An important factor that links all the case studies mentioned earlier is the presence of an infectious trigger. In all the above cases, the outcome has been favourable following a prompt assessment of endocrine status, medical management, and surveillance with imaging modalities. Increased awareness of AH across various settings is crucial to prevent adrenal crisis and, in some cases, underdiagnosis and overtreatment.
